# Monitoring Cerebral Oxygenation in Neonates: An Update

**DOI:** 10.3389/fped.2017.00046

**Published:** 2017-03-14

**Authors:** Laura Marie Louise Dix, Frank van Bel, Petra Maria Anna Lemmers

**Affiliations:** ^1^Department of Neonatology, Wilhelmina Children’s Hospital, University Medical Center Utrecht, Utrecht, Netherlands; ^2^Monash Newborn, Monash Medical Centre, Melbourne, VIC, Australia

**Keywords:** near-infrared spectroscopy, cerebral oxygenation, neonates, neonatal intensive care, neonatal neurology

## Abstract

Cerebral oxygenation is not always reflected by systemic arterial oxygenation. Therefore, regional cerebral oxygen saturation (rScO_2_) monitoring with near-infrared spectroscopy (NIRS) is of added value in neonatal intensive care. rScO_2_ represents oxygen supply to the brain, while cerebral fractional tissue oxygen extraction, which is the ratio between rScO_2_ and systemic arterial oxygen saturation, reflects cerebral oxygen utilization. The balance between oxygen supply and utilization provides insight in neonatal cerebral (patho-)physiology. This review highlights the potential and limitations of cerebral oxygenation monitoring with NIRS in the neonatal intensive care unit.

## Introduction

It has been nearly 8 years, since our research group published a review on the value and pitfalls of cerebral oxygenation monitoring with near-infrared spectroscopy (NIRS) in neonatology (1). Since then, research into cerebral NIRS has taken an impressive flight. The importance of cerebral oxygenation and perfusion monitoring has been increasingly recognized in neonatal intensive care. In this review, the development of cerebral NIRS monitoring over the past years is summarized.

### Value of Cerebral Oxygenation Monitoring

Unfortunately, brain injury is still common in preterm neonates and can lead to a wide range of complications later in life, such as behavioral, attentional, cognitive, sensorimotor or language impairments, and epilepsy (2). Both the increasing number of preterm infants and improved survival rates contribute to the prevalence of neonatal brain injury (3, 4). Preterm infants are particularly susceptible to brain injury as the brain undergoes rapid development during the last trimester of pregnancy. During this period, the brain does not only increase in volume but also undergoes increasing gyri- and sulcification and myelination and improves connectivity (5). Pre-oligodendrocytes and axons mature, the transient subplate neurons appear, and the cerebellum develops and matures. Throughout this process, the brain is using substantial amounts of oxygen (2, 6, 7). Cerebral pathology can present as white matter injury, such as periventricular leukomalacia, or as periventricular–intraventricular hemorrhage (PIVH) (2). Inadequate or fluctuating cerebral perfusion and oxygenation can result in brain injury (8). Hyperoxia, hypoxia, and fluctuations in cerebral oxygenation, indicative of poor cerebral autoregulation, can adversely affect brain development (9–12).

Vital parameters such as blood pressure, heart rate, and pulse oximetry [arterial oxygen saturation (SaO_2_)] are important to assess the condition of the neonate but do not directly assess brain oxygenation (13, 14). NIRS-monitored regional cerebral oxygen saturation (rScO_2_) is a non-invasive and elegant method to monitor global brain oxygenation. rScO_2_ monitoring can be used at bedside for extended periods of time (up to several days) without side effects. Other methods that examine the brain, such as cranial ultrasound or MRI, do not allow for continuous monitoring. NIRS can be used even in the sickest infants and requires minimal handling of the infant. The device can be used at bedside in the NICU as well as during surgery or transportation (15). NIRS can easily be combined with monitoring of cerebral electrical activity by amplitude-integrated electro-encephalography (aEEG).

### NIRS Technique

The NIRS technique is based on the relative transparency of biological tissue to light. Neonatal cerebral tissue can easily be penetrated by NIR light (700–1,000 nm) due to thin overlaying layers of skin and skull. An emitter sends light of the near-infrared spectrum through cerebral tissue in a semi-curved shape to a detector, approximately 2–3 cm in depth (16). Oxygenated (O_2_Hb) and deoxygenated (HHb) hemoglobin absorb the NIR light at different wavelengths, together accounting for total Hb (THb = O_2_Hb + HHb). Differences in NIR light absorption are detected by the sensor and used to calculate the concentrations of Ohb and HHb according to the modified law of Lambert–Beer. The ratio between O_2_Hb and HHb is expressed as the rScO_2_ or tissue oxygenation index (TOI), depending on the manufacturer of the NIRS device. Previous research has shown good correlation between TOI and rScO_2_ (17, 18). The NIR light is absorbed by HHb and O_2_Hb in both arterial and venous vessels, in a 25:75% ratio, and thus NRS reflects mainly cerebral venous oxygen saturation (19). The NIR light is absorbed by both superficial tissues and the cerebral cortex. When two or more detectors are used, the deeper signal reflecting cerebral cortex oxygenation can be distinguished from the superficial signal, reducing the influence of scattering. The technical details of NIRS are beyond the scope of this review but are well described elsewhere (20–23). Most commercially available devices utilize the continuous-wave technique, which measures the attenuation (or absorption) of NIR light from a continuous light source to calculate oxygenation (24). Other NIRS techniques, such as time-resolved spectroscopy and frequency-resolved spectroscopy, are now able to assess cerebral blood volume and quantify absolute concentrations of HHb and O_2_Hb, respectively. However, these techniques have not yet been proved practically useful in neonatal care (24).

### Sensors and Devices

**Figure 1:**
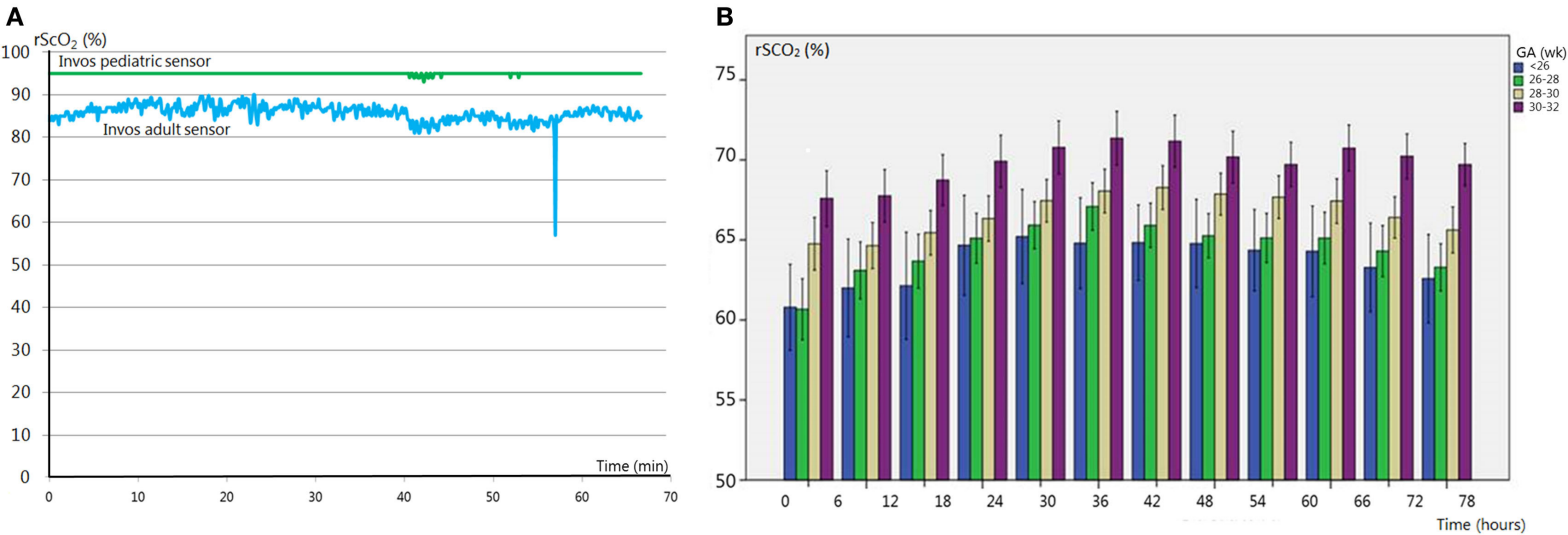
**(A)** Regional cerebral oxygen saturation (rScO_2_) monitored with an adult (blue line) and pediatric (green line) sensor. Hyperoxia values are untraceable with the pediatric sensor due to the cutoff value of 95%. **(B)** Reference values [stratified for gestational age (GA)] of rScO_2_ in premature infants (GA < 32 weeks). Adapted from Ref. (29).

Today, there are several different NIRS devices and sensors commercially available. A number of comparative studies have shown that the overall correlation between NIRS devices is acceptable, although they differ in technique and algorithm (25–27). Smaller and more flexible sensors have been designed for neonatal use. However, these neonatal sensors measure 10% higher compared to the adult sensors (28, 29). Since the upper limit of most devices is set to 95%, high cerebral oxygenation values as measured by the neonatal sensors are shown as a flat line in which all variation is lost, as demonstrated in Figure 1A. NIRS device and sensor type must be taken into account when NIRS monitoring of cerebral oxygenation is applied in clinical care. Reference values for the neonatal sensor have been published (see below) (29).

### Validation

Regional cerebral oxygen saturation represents a mixed saturation largely determined by the venous component (75%), which is why NIRS validation studies have often focused on venous saturation (19). However, venous saturation does not reflect mixed arterial and venous saturation as NIRS does, and there is no “gold standard” to measure venous oxygen saturation (30). A good correlation has been reported between oxygen saturation in the jugular vein and NIRS-monitored cerebral oxygenation, with a mean difference of 5%, for different manufacturers (Hamamatsu, INVOS, CAS-MED) (31–34). However, the difference between jugular venous oxygen saturation and regional cerebral oxygenation may increase during hypoxia. This is presumably caused by an increased arterial contribution to the NIRS signal due to cerebral arterial vasodilatation as a response to hypoxia (35). Cerebral fractional tissue oxygen extraction (cFTOE) has been validated against central cerebral venous saturation in newborn piglets (36). Brain perfusion assessment with NIRS has been compared to perfusion assessment with MRI, which has shown strong correlations (37, 38). Both rScO_2_ and TOI have shown good reproducibility (39, 40).

### Reference Values

Several studies have analyzed changes in rScO_2_ with advancing postnatal age. rScO_2_ is between approximately 40 and 56% directly after birth (irrespective of delivery mode) (41–43), increases up to 78% in the first 2 days after birth (44) and then slowly stabilizes during 3–6 weeks after birth with values between 55 and 85% (45–47). Several studies have published reference ranges immediately after birth, which show a gradual increase during the first 15 min of life (42, 46). A recent study by Alderliesten et al. provides reference values based on a large study cohort during the first 72 h of life in preterm infants [<32 weeks gestational age (GA); *n* = 999]. The data are converted into reference curves stratified for different GAs which can be used for cot side interpretation of rScO_2_ and cFTOE values, as shown in Figure 1B (29). These reference values, obtained with the (small) adult sensor (SomaSensor SAFB-SM, Covidien, Mansfield, MA, USA), will facilitate clinical application of cerebral oxygenation monitoring. As stated above, it is important to realize that neonatal sensors of various NIRS manufacturers display higher values (up to 10%) as compared to adult sensors (28).

## Clinical Application

Gaining insight into the oxygenation of the neonatal brain can be of important clinical value, as a large share of neonatal pathology is brain associated. NIRS monitoring of cerebral oxygenation can be considered in several clinical situations as outlined below.

### Cerebral Oxygenation and the Patent Ductus Arteriosus

**Figure 2:**
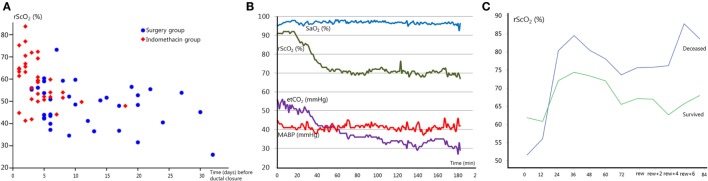
**(A)** Regional cerebral oxygen saturation (rScO_2_) just before ductal closure in patients treated with indomethacin (red squares) or surgery (blue circles) as a function of postnatal age in days. Note that the majority of infants requiring surgical treatment are exposed to the lowest rScO_2_ values for a longer period. Adapted from Ref. (14). **(B)** Acute end-tidal CO_2_ (etCO_2_) decrease results in a subsequent reduction in rScO_2_, on the contrary arterial oxygen saturation (SaO_2_) remains stable. MABP, mean arterial blood pressure. **(C)** rScO_2_ during hypothermia and after rewarming (rew) in two severely asphyxiated infants. The infant with an adverse outcome (blue line) showed higher rScO_2_ values compared to the infant that survived (green line). Cerebral fractional tissue oxygen extraction values (not shown) mirrored rScO_2_ values.

The hemodynamically significant patent ductus arteriosus (PDA) remains a controversial topic. Clinicians and researchers are still debating whether or not it should be treated, what the best treatment strategy is and when would be the best time to intervene (48–51). Unfortunately, the brain is rarely included in this discussion. A PDA can negatively influence cerebral oxygenation. Shunting of the blood through the duct away from the brain has a profound negative effect on rScO_2_. This effect is independent from SaO_2_, which remains within normal limits during a PDA (13, 52). Cerebral oxygenation normalizes after ductal closure (13, 52). The ductal diameter is associated with cerebral oxygenation, where a larger diameter (indicating a significant left to right ductal shunt) is associated with lower rScO_2_ (52). Infants who need surgical PDA closure are often exposed to low rScO_2_ values for a longer period of time, as shown in Figure 2A, and are therefore at risk of cerebral injury (14). Additionally, a further reduction in cerebral oxygenation occurs during ductal surgery (53, 54). Weisz et al. reported an increased risk of neurodevelopmental impairment in infants after surgical ductal ligation compared to pharmaceutically treated infants (55). More specifically, underdevelopment of the cerebellar structure has been reported in infants who needed surgical closure (14). Extended episodes of low cerebral oxygenation are most likely responsible for this phenomenon (14).

### Cerebral Oxygenation and Respiration

Preterm infants often require respiratory support, which can affect cerebral hemodynamics and cerebral oxygenation (56, 57). An earlier study reported NIRS-monitored changes in cerebral blood flow (CBF) during continuous positive airway pressure and artificial ventilation. CBF significantly correlated with the type of respiratory support, leading to the conclusion that ventilation can impact cerebral circulation (58). Cerebral oxygenation can also be affected by the type of ventilation support during surgery (59).

Ventilation is the main regulatory mechanism of arterial carbon dioxide pressure (pCO_2_). pCO_2_ can affect the brain by altering cerebral arterial vessel diameter, where hypercapnia can induce cerebral vasodilatation and hypocapnia induces vasoconstriction (60). As such, pCO_2_ can affect cerebral perfusion and oxygenation, and both hyper- and hypocapnia have been associated with neuropathology (61, 62). An increase in pCO_2_ is accompanied by an increase in cerebral oxygen saturation with a decrease in oxygen extraction (63, 64). Acute fluctuations in pCO_2_, even within the normal range, appear to directly affect the neonatal brain perfusion (personal communication). The pCO_2_-induced changes in cerebral perfusion and oxygenation can be monitored by NIRS, as shown in Figure 2B, in order to identify and prevent pCO_2_-induced cerebral hypo- or hyperperfusion and brain damage.

Other factors related to respiration have also been shown to influence cerebral oxygenation. Apneas, for example, can affect brain oxygenation, and high mean airway pressure during artificial ventilation can also reduce cerebral oxygenation (1, 65–67). Also, infants with respiratory distress syndrome (RDS) have lower cerebral oxygenation and increased variance in rScO_2_ and cFTOE during the first 3 days after birth (68, 69). Moreover, they often have an impaired cerebral autoregulation, which may further predispose them to cerebral injury (70). Combining arterial blood pressure and cerebral oxygenation measures can help to identify (lack of) cerebral autoregulation (see below).

### Cerebral Oxygenation and Autoregulation

Cerebral autoregulation is the ability to maintain stable cerebral perfusion and oxygenation during fluctuations in blood pressure (71). Hypotension can cause a severe reduction in cerebral perfusion and impairment of cerebral autoregulation, leading to inadequate perfusion (72). Combining rScO_2_-monitoring with arterial blood pressure monitoring enables assessment of cerebral autoregulation (73). Prematurity is a risk factor for impaired autoregulation, and even small variations in blood pressure can affect cerebral oxygenation in clinically sick and unstable babies (48, 74). Cerebral autoregulation can indeed be affected in several clinical situations that are commonly seen in preterm infants. Our group has previously demonstrated that RDS predisposes for lack of cerebral autoregulation (70). Autoregulation might also be impaired during surgery and high concentrations of positive inotropes such as dopamine (11, 49). Impaired autoregulation has been linked to poor neurodevelopmental outcome (50). Evaluating cerebral autoregulation at bedside can identify episodes of impaired autoregulation, and appropriate measures can be initiated to stabilize cerebral perfusion and oxygenation. Cerebral autoregulation can be computed in different ways, and software to calculate autoregulation at bedside is currently being developed (51). In summary, cerebral autoregulation may become impaired, especially in the unstable (preterm) infant, predisposing these neonates to brain injury. This underlines the importance of cerebral oxygenation an autoregulation monitoring in the early neonatal period (75).

### Cerebral Oxygenation and Hypotension

Cerebral oxygenation can play an important role in assessing hypotension and whether positive inotropic therapy is indicated. There is an increasing awareness that the current definitions of *hypotension of prematurity* do not always reflect true hypotension. Permissive hypotension is increasingly accepted, unless there are (clinical) signs of hypoperfusion (76–78). As already stated above, hypotensive treatment is not without side effects and may have adverse effects on outcome (11, 79). Cerebral oxygenation plays an important role as a marker of end-organ oxygenation and can help making decisions whether or not treatment for hypotension is indicated. Other parameters such as blood gasses, urine production, and capillary refill should be taken into account. Identifying small reductions in blood pressure that do not affect cerebral oxygenation, and systemic perfusion might prevent unnecessary treatment with inotropes (78, 80). Monitoring rScO_2_ and cerebral autoregulation during neonatal surgery is important to prevent hypotension-related injury to the immature brain (see also below) (81). Our research group is currently involved in a prospective study (the TOHOP study) to find an answer to the question at which stage hypotension treatment is warranted (TOHOP; http://ClinicalTrials.gov identifier: NCT01434251).

### Cerebral Oxygenation and Small-for-Gestational-Age (SGA) Neonates

Preterm infants who are born SGA show higher cerebral oxygenation during the first postnatal days (29, 82). This is most likely related to the prenatal blood flow redistribution of the intrauterine growth restricted (IUGR) fetus, in an attempt to preserve oxygen supply to the brain (brain sparing effect) (83). However, this does not necessarily protect against cerebral injury, and infants born following IUGR are at an increased risk of neurodevelopmental impairment (84, 85). In case of a PDA, SGA infants demonstrated a significantly larger fall in cerebral oxygenation, as compared to AGA infants (86).

### Cerebral Oxygenation and Neurodevelopmental Outcome

Disturbances in cerebral perfusion and oxygenation are major contributors to neonatal brain injury, increasing the risk of impaired neurodevelopmental outcome (8, 87). Infants are particularly susceptible to brain injury during the first 3 days after birth, when major hemodynamic transitional changes occur. A large international randomized controlled trial, the SafeboosC study (Safeguarding the brains of out smallest children), has investigated whether it is possible to reduce the hypoxic and/or hyperoxic burden on the immature brain with cerebral oxygenation monitoring, in order to prevent neurological damage and to improve outcome (88). The study has shown that disturbances in cerebral oxygenation could be identified with NIRS. A treatment protocol prescribed treatment steps to restore normal brain oxygenation. The burden of hypoxia (and hyperoxia), as expressed by the percentage of time spend outside the normal range of rScO_2_ (55–85%), was significantly lower in the group with (visible) NIRS monitoring as compared to the blinded control group (median 36.1 vs. 81.3%) (47). This difference was mainly due to a reduction in hypoxic episodes.

Impaired cerebral oxygenation below the threshold of 55% appears to affect neurodevelopmental outcome at 15 and 24 months corrected age (personal communication). Poor cerebral autoregulation, examined by the correlation between rScO_2_ and arterial blood pressure, has been be associated with an increased risk score predictive of neonatal mortality and morbidity (CRIB II) (89).

Several studies, in newborn animals and humans, showed that rScO_2_ values consistently below 40% (measured with adult sensors) are related to brain damage (90–92). Other clinical studies showed that low cerebral oxygen saturation immediately after birth (<15 min) is associated with PIVH (93). In accordance with these results, low cerebral oxygenation during the first 48 h after birth was associated with death or severe PIVH in a study by Cerbo et al. (94). Similarly, increased oxygen extraction cFTOE can precede development of PIVH (95, 96).

### Cerebral Oxygenation and Red Blood Cell Transfusions

Several studies have shown a significant increase in cerebral oxygenation after red blood cell transfusions in anemic infants (97, 98). The infants with the lowest pre-transfusion rScO_2_ values seem to benefit the most from transfusions (99). Similarly, high cFTOE levels (>0.4) can indicate an imbalance between cerebral oxygen supply and demand, which may underline the need for red blood cell transfusion (100). This indicates that cerebral oxygenation monitoring might be useful as a marker to identify infants with high cFTOE and/or low rScO_2_ who might benefit from blood transfusions (100–102).

### Cerebral Oxygenation and Neonatal Surgery

Infants with cardiac or non-cardiac anomalies may require major surgery in the first few months after birth (103). Exposure to neonatal surgery can put the immature brain at risk (104, 105). An increased risk of neurodevelopmental delay after neonatal surgery has indeed been reported (106, 107). Both the procedure as well as anesthetics can be harmful (108–110). Monitoring cerebral oxygenation during surgery to increase cerebral safety is therefore advised (111–116). Perioperative monitoring evaluates brain oxygenation pre- and postsurgery, while intraoperative monitoring can assist surgeons and anesthesiologists to optimize cerebral oxygenation during the procedure to protect the neonatal brain (113, 117, 118). During surgery, cerebral NIRS can detect episodes of hypoxia more reliably than arterial SaO_2_ monitoring (114, 119). Introduction of cerebral oxygenation monitoring during cardiac surgery has improved intraoperative transfusion management (120). Cerebral oxygenation monitoring can also reflect changes in vital parameters during cardio-pulmonary bypass (121).

### Cerebral Oxygenation and Hypoxic-Ischemic Encephalopathy (HIE)

Previous studies have demonstrated that rScO_2_ is increased and cFTOE is decreased during the first days after severe birth asphyxia, and these findings have been correlated with an adverse outcome at 2 years of age (Griffiths Mental Developmental scales) (122, 123) (see also Figure 2C). NIRS monitoring combined with simultaneous assessment of aEEG background patterns has a strong prognostic value for long-term neurodevelopmental outcome. High cerebral oxygenation with an abnormal aEEG background pattern (low electrical activity) in severely asphyxiated neonates with hypothermia treatment at 12 h of age has a positive predictive value of 91%, absence of these results in a negative predictive value of 100% (123). These findings strongly suggest that NIRS monitoring of cerebral oxygenation can have an important role in the (early) prognosis of neurodevelopmental outcome. Cerebral hyperoxygenation in neonates with an adverse outcome is most likely explained by low energy metabolism after severe brain injury with low oxygen utilization, cerebral hyperperfusion, and impaired autoregulation of the cerebral vascular bed (124, 125). These findings have been confirmed in other studies, incorporating MRI (126). Cerebral oxygenation with NIRS correlates strongly with CBF as assessed by arterial spin labeled MRI in infants with severe HIE (37).

## Limitations

Hair, dark skin, and interfering light from other sources such as phototherapy devices can pose a problem during NIRS monitoring (1). Subdural edema or hematoma below the sensor might also interfere with measurements (127) in small infants, placement of the electrode might be challenging if they also require simultaneous aEEG monitoring. The curvature of the skull and head circumference has been mentioned as potential limitations (24). However, Alderliesten et al. did not find a correlation between head circumference and rScO_2_, stating that influence of head curvature seems unlikely (29). As previously discussed, type of NIRS device and sensor must be taken into account when interpreting cerebral oxygenation values (28).

## Conclusion

Injury to the immature brain remains a major contributor to neonatal mortality and morbidity. Monitoring vital parameters provides us with critical information concerning the condition of the infant but does not offer direct information regarding brain oxygenation and perfusion. Cerebral oxygenation monitoring with NIRS, at least during the vulnerable transition period throughout the first 3 days after birth, provides the clinician with additional important information. Several clinical conditions can affect brain oxygenation, and studies have shown that systemic oxygen saturation does not always reflect cerebral oxygenation. The assessment of neonatal brain oxygenation (and perfusion) can be extremely useful in the clinical setting. It has the potential to guide clinical management in order to prevent brain injury and to avoid unnecessary treatment. It may also provide important information regarding the infant’s prognosis.

## Author Contributions

LD determined review subjects, collected, analyzed, and included the literature, drafted the initial manuscript, and approved the final manuscript and is accountable for all aspects of the manuscript. FvB and PL determined review subjects, interpreted the literature, reviewed and revised the manuscript, and approved the final manuscript and are accountable for all aspects of the manuscript.

## Conflict of Interest Statement

The authors declare that the research was conducted in the absence of any commercial or financial relationships that could be construed as a potential conflict of interest.

## Abbreviations

aEEG, amplitude-integrated electro-encephalography; cFTOE, cerebral fractional tissue oxygen extraction; NIRS, near-infrared spectroscopy; PDA, hemodynamically significant patent ductus arteriosus; PIVH, periventricular–intraventricular hemorrhage; RDS, respiratory distress syndrome; rScO_2_, regional cerebral oxygen saturation; SaO_2_, arterial oxygen saturation; TOI, tissue oxygenation index.
