# Corrigendum: Monitoring Cerebral Oxygenation in Neonates: An Update

**DOI:** 10.3389/fped.2017.00160

**Published:** 2017-07-21

**Authors:** Laura Marie Louise Dix, Frank van Bel, Petra Maria Anna Lemmers

**Affiliations:** ^1^Department of Neonatology, Wilhelmina Children’s Hospital, University Medical Center, Utrecht, Netherlands; ^2^Monash Newborn, Monash Medical Centre, Melbourne, VIC, Australia

**Keywords:** near-infrared spectroscopy, cerebral oxygenation, neonates, neonatal intensive care, neonatal neurology

Cerebral Oxygenation and the Patent Ductus Arteriosus

The hemodynamically significant patent ductus arteriosus (PDA) remains a controversial topic. Clinicians and researchers are still debating whether or not it should be treated, what the best treatment strategy is and when would be the best time to intervene (48–51).

Reference 48–51 are:

48. Tsuji M, Saul JP, du Plessis A, Eichenwald E, Sobh J, Crocker R, et al. Cerebral intravascular oxygenation correlates with mean arterial pressure in critically ill premature infants. *Pediatrics* (2000) 106(4):625–32. doi:10.1542/peds.106.4.625
49. Brady KM, Mytar JO, Lee JK, Cameron DE, Vricella LA, Thompson WR, et al. Monitoring cerebral blood flow pressure autoregulation in pediatric patients during cardiac surgery. *Stroke* (2010) 41(9):1957–62. doi:10.1161/STROKEAHA.109.575167 PMID:20651273
50. Wong FY, Leung TS, Austin T, Wilkinson M, Meek JH, Wyatt JS, et al. Impaired autoregulation in preterm infants identified by using spatially resolved spectroscopy. *Pediatrics* (2008) 121(3):e604–11. doi:10.1542/peds.2007-1487 PMID:18250118
51. Caicedo A, Alderliesten T, Naulaers G, Lemmers P, van Bel F, Van Huffel S. A new framework for the assessment of cerebral hemodynamics regulation in neonates using NIRS. *Adv Exp Med Biol* (2016) 876:501–9. doi:10.1007/978-1-4939-3023-4_63 PMID:26782251

However, these references are supposed to be:

– Perez KM, Laughon MM. What is new for patent ductus arteriosus management in premature infants in 2015? *Curr Opin Pediatr* (2015) 27(2):158–64. doi:10.1097/MOP.0000000000000200
– Evans N. Preterm patent ductus arteriosus: a continuing conundrum for the neonatologist? *Semin Fetal Neonatal Med* (2015) 20(4):272–7. doi:10.1016/j.siny.2015.03.004 PMID:25818393
– Ibrahim TK, Haium AA, Chandran S, Rajadurai VS. Current controversies in the management of patent ductus arteriosus in preterm infants. *Indian Pediatr* (2014) 51(4):289–94. doi:10.1007/s13312-014-0403-2 PMID:24825266

The authors apologize for this error and state that this does not change the scientific conclusions of the article in any way.

## Conflict of Interest Statement

The authors declare that the research was conducted in the absence of any commercial or financial relationships that could be construed as a potential conflict of interest.

